# *PlateEditor*: A web-based application for the management of multi-well plate layouts and associated data

**DOI:** 10.1371/journal.pone.0252488

**Published:** 2021-05-28

**Authors:** Vincent Delorme, Minjeong Woo, Virginia Carla de Almeida Falcão, Connor Wood

**Affiliations:** Tuberculosis Research Laboratory, Institut Pasteur Korea, Seongnam, Gyeonggi, Republic of Korea; Universidad Internacional de La Rioja, SPAIN

## Abstract

Multi-well plates are convenient tools to work with in biology experiments, as they allow the probing of multiple conditions in a compact and economic way. Although both free and commercial software exist for the definition of plate layout and management of plate data, we were looking for a more flexible solution, available anywhere, free from download, installation and licensing constraints. In this context, we created *PlateEditor*, a free web-based, client-side application allowing rapid creation of even complex layouts, including dose-response curves and multiple combination experiments for any plate format up to 1536 wells. *PlateEditor* also provides heatmap visualization and aggregation features to speed-up the process of data analysis and formatting for export in other application. Written in pure JavaScript, it is fully open-source, can be integrated in various workflows and has the potential to be extended with more functionalities in the future.

## Introduction

Multi-well plates offer a convenient and compact way to test multiple conditions in one go. From 6 to 1536 wells, made with various materials (plastic, glass) and for various purposes (optical measurements, filtration, storage), they can accommodate a broad range of assays and conditions. The greater availability of automation equipment, for example readers, washers and incubators, allows easy upscaling from a couple of plates to a full library of hundreds of plates. Screening activities have a heavy reliance on multi-well plates, but other common applications like enzyme-linked immunosorbent assays (ELISA) or dose-response curves are also commonly performed in multi-well plates. Most of the automation or plate reading software use or generate data files as lists, using the well name or row/column indices to locate the values at their relevant location. While this is ideal from an automation point-of-view our experience shows that such files are uncomfortable to read and work with for a regular lab worker, who will prefer a more visual, plate-like (or matrix-like) representation of the data, usually under the form of heatmap.

Every plate reader or plate-friendly device will propose such a tool to prepare plate layouts and analyze their associated data. Other commercial solutions exist for the creation and visualization of plate data (*Pipeline Pilot*, *Spotfire* and *Dotmatics*, among others). All of them are locked behind high licensing fees and may not be available to install on the individual computers of multiple researchers, or require connection to local databases, making them hardly available offsite. For us, such a feature has however become more and more desirable, precipitated by the current COVID epidemic and the constraints of home-working, which are likely also experienced by other researchers across the world. The possibility of exchanging layout data from lab to lab are also impaired by the proprietary format chosen by each vendor: layouts from different software are usually not compatible, or will require some form of post-processing before being usable elsewhere. Although free solutions are available for plate management, few offer a ready-to-use application (*i*.*e*. provided under the form of libraries) or were designed for or within specific applications. Some examples of free plate management systems featuring a plate layout editor include: *Brunn* [1], *KNIME* [2], *AutoLabDB* [3] and *SLIMS* [4]). The Java application *DMAN* also includes a layout editor [5], but is dedicated to differential scanning fluorimetry experiments. The *JavaScript Plate Layout* application is also freely available, but is provided as a library and limited to 96-well plates (https://github.com/nebiolabs/plate-map). Specifically, we were interested in an application that could cover all of the following features: *i*) free, ready and easy-to-use (visual) editor for definition of layouts for multi-well plates of any format, up to 1536-well plate; *ii*) web-based application, accessible anywhere from any device, free from module installation or need to download updates; *iii*) able to guarantee strict data confidentiality; *iv*) allowing basic but flexible data visualization and aggregation features.

To our knowledge there are currently no free applications reported that could cover all these needs. To cover this gap and provide additional tools for scientists working with multi-well plates, we developed *PlateEditor* and present here its main features and some usage examples. The application can be accessed at the following address: https://plateeditor.sourceforge.io/.

## Materials & methods

### Source code

*PlateEditor* was written in pure JavaScript (JS) and uses only two external libraries as dependencies: *PapaParse*, for client-side parsing of text and csv files (v5.0; https://www.papaparse.com) and *JSZip*, for reading and writing of zip archives (v3.2.0; https://stuk.github.io/jszip). The source code is divided into four independent packages: *editor*.*min*.*js* regroups the classes needed by the plate layout editor; *analyzer*.*min*.*js* regroups the classes needed for data aggregation (including the Z-score calculation); *shared*.*min*.*js* regroups the classes needed for the file parsing, mapping and plate pairing; *ui*.*min*.*js* regroups the classes needed for the user interface. All classes and functions were written as independent JS files, organized in four folders named after the package to which they belonged (editor, analyzer, shared, ui). The packages were built by merging together all files from a single folder and minifying the resulting file, using the JS library Gulp (v4.0.0; https://gulpjs.com/). The cascade style sheet (CSS) files used by the web application were written and packaged the same way (merged and minified using Gulp). Each JS package file has an associated CSS package file (*editor-styles*.*css*; *analyzer-styles*.*css*, *shared-styles*.*css* and *ui-styles*.*css*). To simplify the development and bug-tracking process, source maps were also generated with Gulp, allowing the initial, non-minified code to be referenced in the browser console upon code execution errors. The application was designed using Firefox Developer Edition (v85; https://www.mozilla.org) and tested with Google Chrome (v87) and Microsoft Edge (v87). The source code is available under the MIT license on the *PlateEditor* repository on GitHub (https://github.com/vindelorme/PlateEditor) and SourceForge (https://sourceforge.net/projects/plateeditor). The latest version of the full application, as well as the version available at the time of the manuscript review, can both be downloaded as zip archives on SourceForge or GitHub.

### Statistics

For a population of n discreet numerical values, whose average is equal to *Avg*, the standard deviation (SD) and coefficient of variation (CV, %) of the population were calculated as follows:

$$SD=\sqrt{\frac{1}{n}\sum_{i=0}^{n}{\left(x_{i}-Avg\right)}^{2}}$$

$$CV=100\times \frac{SD}{Avg}$$

The formula used for calculation of the Z-factor (Z’, [6]) and window (W) are as follows:

$$Z^{\prime }=1-\frac{3\times ({SD}_{pos}+{SD}_{neg})}{\left|{Avg}_{pos}-{Avg}_{neg}\right|}$$

$$W=\left\{ \begin{array}{c} \frac{{Avg}_{pos}}{{Avg}_{neg}}\;if\;{Avg}_{pos}\geq {Avg}_{neg}\\ \frac{{Avg}_{neg}}{{Avg}_{pos}}\;if\;{Avg}_{neg}\geq {Avg}_{pos} \end{array}\right.$$

Where *Avg*_*pos*_, *SD*_*pos*_, *Avg*_*neg*_, *SD*_*neg*_ are respectively the average and standard deviation of the aggregated values for the positive and negative controls.

### Reproducibility

Multiple data files of various format and origins were used for the validation of the algorithms. Real-case files generated automatically by plate readers or other applications were included, along with manually created files containing randomized data. The source code includes test files used for the testing and validation of the application (*tests* folder). For each file, a short description of its usage and expected output is given in the Readme file present in this folder. Both in the source code and the bundled application, other files used for demonstration purposes are also included (*examples* folder). The files used to generate the figures and examples described in this manuscript are all available in this folder.

## Application overview

**Fig 1:**
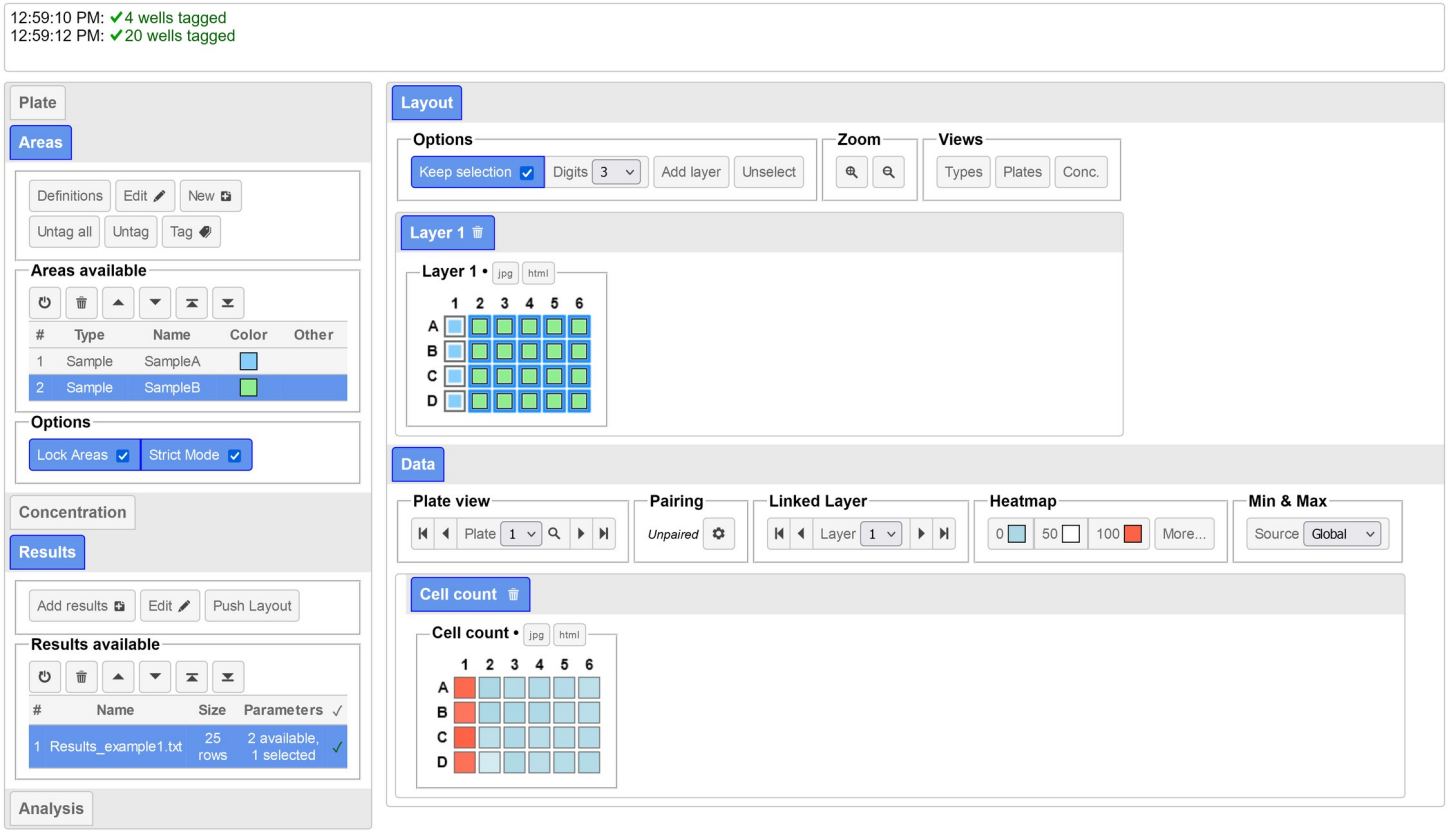
Overview of *PlateEditor*. Top: console panel, in which feedback messages are displayed. Left: menu panel, includes all the controls and options for management of the layout, creation and tagging of areas, loading of result and definition files, as well as the analysis tools. Right: main section, divided into two panels; the *Layout* panel on top is used to select wells and tag the Areas. The *Data* panel at the bottom allows visualization of the data from the selected result files as heatmaps. This figure can be reproduced using the files from the *Simple* example.

*PlateEditor* allows the creation of *Areas*, which can be added to the desired wells in a process referred to as tagging (**Fig 1**). Wells can be selected by drawing a selection box (or lasso) on the plate. New wells can be added to the selection by maintaining the *Ctrl* key pressed. Entire columns or rows can also be selected by clicking or selecting the relevant column/row headers. When different areas need to be tagged in the same well, additional *Layers* can be created and tagged independently to create overlaps. Each area must have a unique name identifying it, and can be of any of the following type: sample (or range), positive control or negative control. The type chosen for the area defines specific rules for the tagging and data aggregation. In particular, control areas cannot overlap with sample or range areas in the default tagging mode (strict mode). Only values for wells tagged with a control will be used by the *Controls* analysis tool.

Ranges are specifically designed to help with repeating sequence of samples. They possess self-incrementing features and can be linked to *Definitions*, data files used for the resolution of the names for each index. This greatly simplifies the process of tagging as only one Area needs to be defined and tagged, instead of a multitude of them. A typical example is a plate layout for primary screening, where each individual well contains a different compound. The plate layout can be defined by tagging a single range in the relevant wells and the name resolution performed automatically through a *Definition* file attached to the range (see Usage examples below).

**Fig 2:**
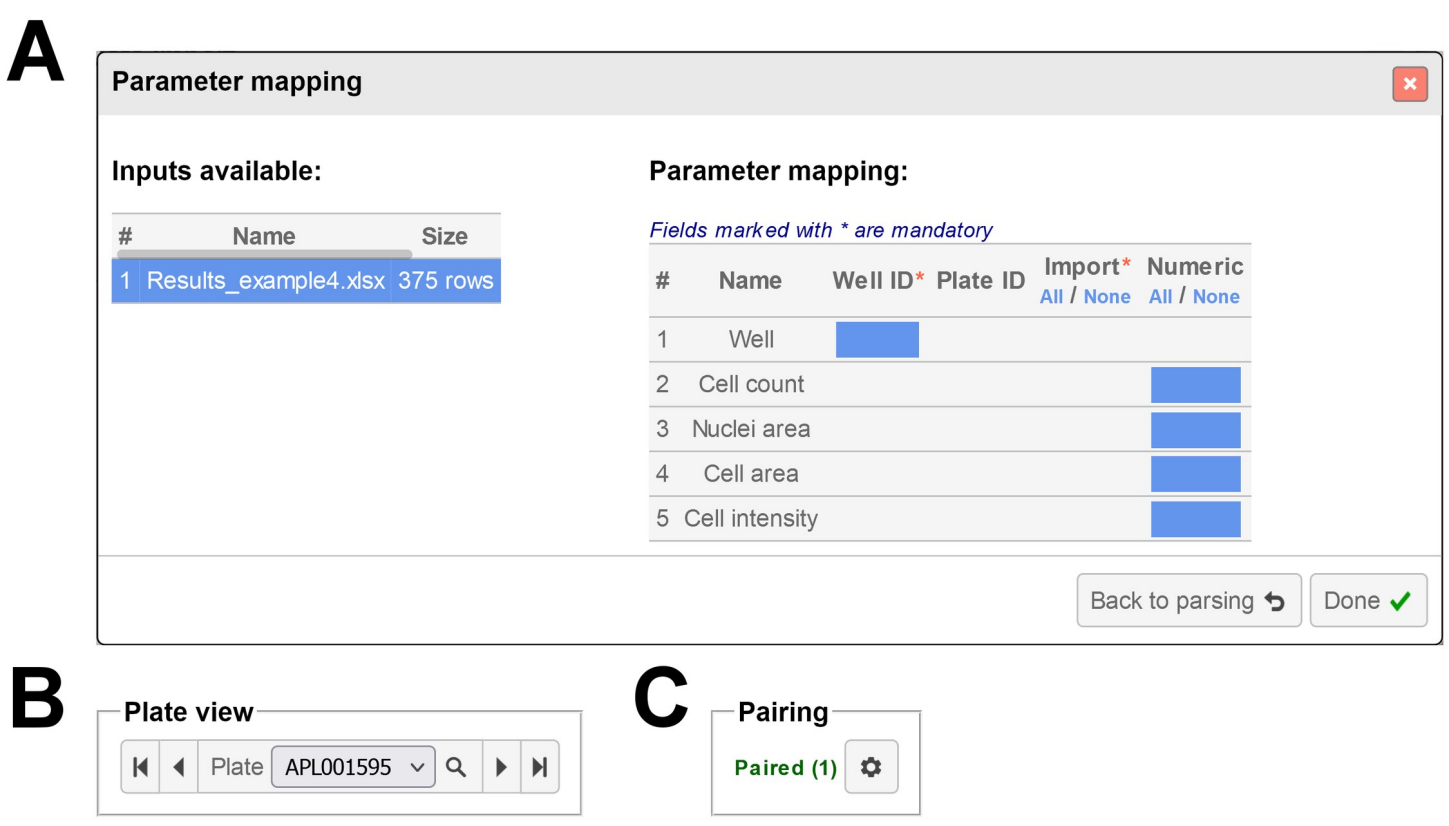
Parameter mapping, plate view and pairing. **A**. Overview of the parameter mapping process for a result file including 5 columns (Well, Cell count, Nuclei area, Cell area, Cell intensity). Any column name including the keyword “well” is automatically assigned to the *Well ID* parameter. An optional *Plate ID* parameter can also be mapped when relevant (multiple plates per file). At least one column should be mapped as a parameter (*Import*); the values for this column will be displayed as heatmap under the *Data* panel of *PlateEditor*. The first row of data is used to automatically determine if the values are numerical or textual, but the user can adjust this setting manually (*Numeric*). **B**. The *Plate View* control allows selection of the plate name or plate index to display for the result file currently selected. Description of the controls, from left to right: display the first plate available; display previous plate; selection of a plate directly from the drop-down list; lookup tool to search specific plate names; display the next plate; display the last plate available. **C**. Pairing indicates whether the selected result plate is paired with one or more definition plates. Pairing options can be adjusted by clicking the gear icon.

Data files can be attached to the layout (*Results*) and the values visualized in the form of 3-color heatmaps. After parsing, a column mapping step is required to indicate which columns contain the data to be visualized, referred to as *Parameters* (**Fig 2A**). The column containing the well name is also required, to allow binding of the data to the relevant wells. Optionally a plate name or plate index column can also be mapped, allowing the visualization of multiple plates within a single result file. For clarity results for only one plate are displayed at a time, but the user can navigate between the available plates using the *Plate View* control (**Fig 2B**), located under the *Data* panel. A pairing tool is available to synchronize the result plates with available definition plates. This allows browsing of the result plates while maintaining a link to the correct range definitions (**Fig 2C**). The user can configure the colors used for the heatmaps using a set of available templates or choosing specific colors among a list of 138. The normalization can also be tuned to use the minimum and maximum values found across all the available plates (default setting), the current plate only, or custom values entered by the user.

**Fig 3:**
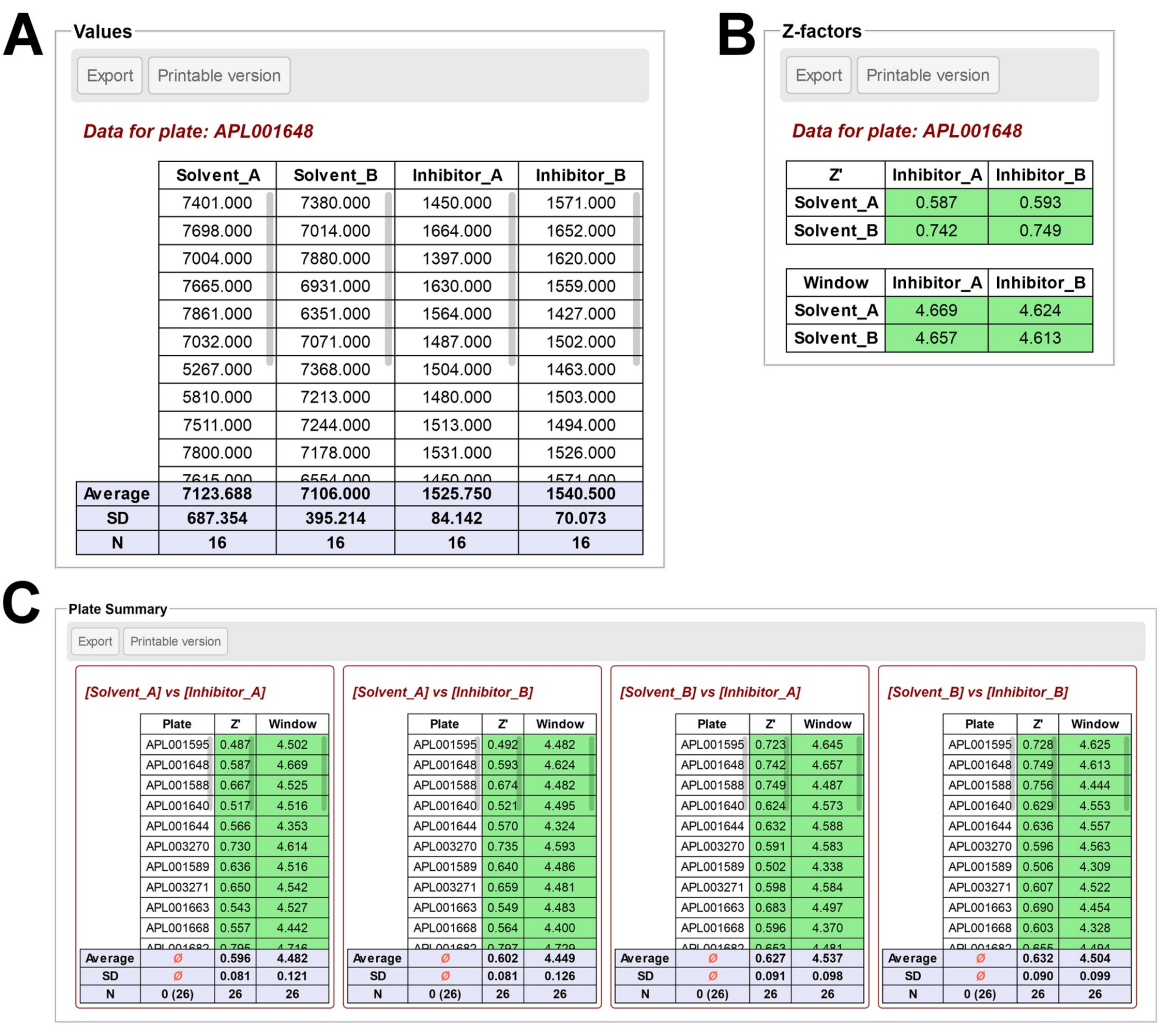
**Overview of the *Controls* analysis tool outputs, for a layout including two positive (Inhibitor_A, Inhibitor_B) and two negative (Solvent_A, Solvent_B) controls. A**. Individual values for each control are aggregated in columns, with their average and standard deviation (SD) computed. N indicates the number of values. **B**. Calculated Z-score (Z’) and Window for all combinations of positive and negative controls (as double-entry tables) for the plate currently selected. **C**. Summary values (Z’, Window) for all visited plates are aggregated in columns, with their average and standard deviation (SD) computed. N indicates the number of plates visited. Values for each positive/negative control combination are indicated in separated tables. This figure can be reproduced using the files from the *Screening* example.

**Fig 4:**
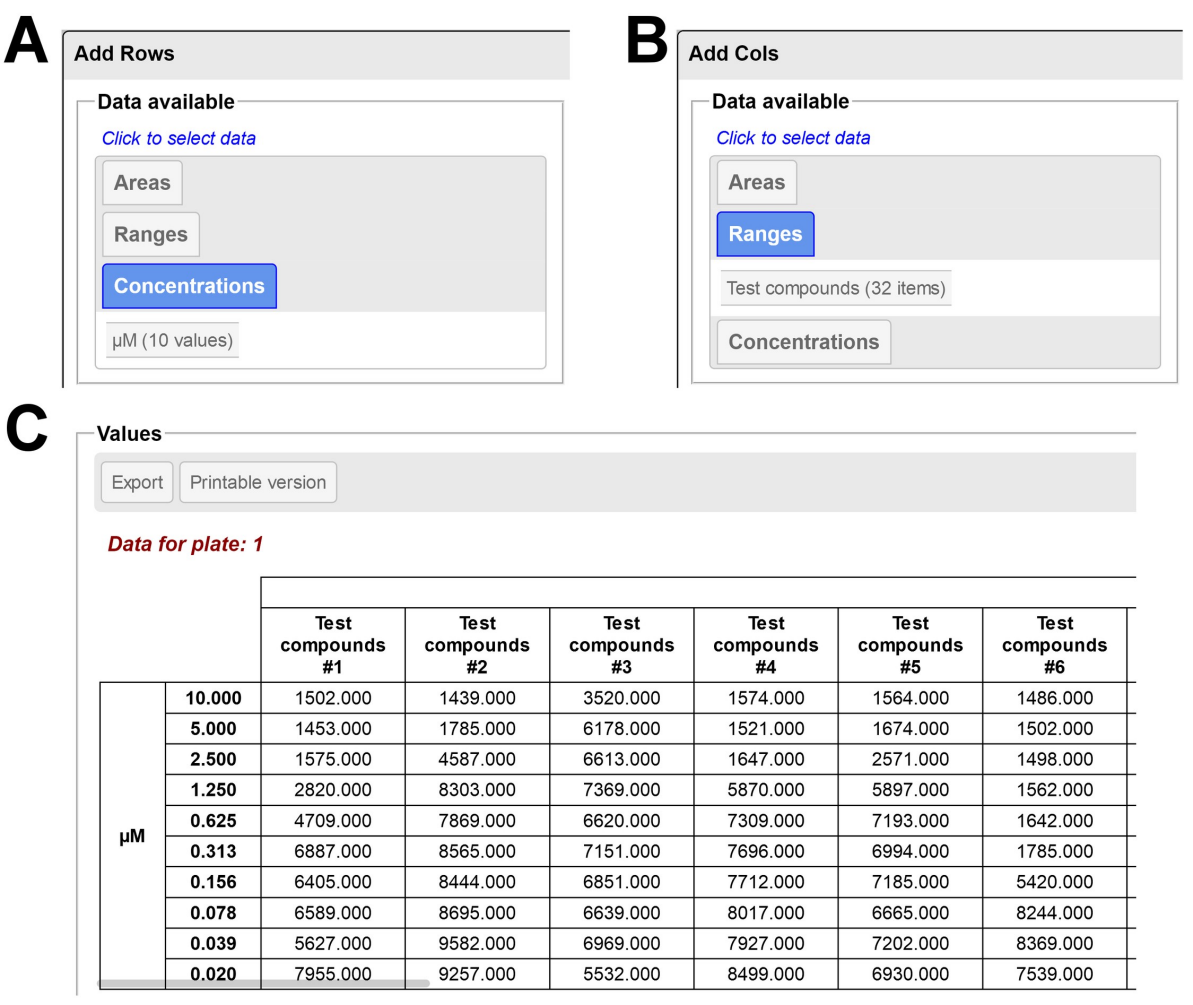
Overview of the Grouped Analysis tool output, for a layout including a range (Test compounds) and multiple concentrations (μM) arranged in dose-response. **A**. For the rows, the concentration series was selected. **B**. For the columns, the range was selected. **C**. Resulting two-entry table obtained using configuration as in **A** and **B**, with the aggregation mode set on *Average* (in this mode, only the average value is shown). The table has 32 columns of data but was truncated here for clarity. This figure can be reproduced using the files from the *DRC* example.

Three analysis tools are available for data aggregation and export. The *Controls* tool aggregates the values for positive and negative controls, calculates the Z-factor (Z’) / Window for all combinations of positive and negative controls available and records these values for each visited plate (**Fig 3**). The *Column Analysis* tool prepares columns of data with statistics (average, standard deviation, N; identical to the table in **Fig 3A**) for each unique combination of sample/range/controls/concentration available on the layout. The coefficient of variation (CV, %) can also be displayed if desired. The *Grouped Analysis* tool allows aggregation of data as a double-entry table, giving the user full control over what to display as Rows and Columns (**Fig 4**). For all analysis tools, individual tables can be downloaded as tab-separated text files or visualized separately in a new window, for printing or easier copy-pasting operations. All tables can also be downloaded at once as an organized zip archive containing all the individual files.

For a more thorough description of all the functionalities of *PlateEditor* and their usage the reader is invited to consult the Wiki pages of the application at the following address: https://sourceforge.net/p/plateeditor/wiki/Home.

## Technical details on key features

### File streaming

In *PlateEditor* the parsed contents of data files attached to the layout, which includes both Results and Definitions files, are not stored in the memory. Although such approach would be acceptable for files of few hundreds of kilobytes (kB), memory limitations would prevent files of several megabytes (MB) or more from being successfully loaded by the web browser, leading it to stall and eventually crash. To address these issues and allow the user to work with potentially big files, a streaming approach allowing the files to be read row-by-row was implemented. The JS library *PapaParse* (https://www.papaparse.com) was directly available for client-side streaming of text or csv files, but no counterparts were available for spreadsheet files (in particular.xls and.xlsx). These spreadsheets can always be converted to text or csv files by the user, so that the *PapaParse* library is theoretically sufficient. In practice, the Microsoft Excel file format is widespread in research laboratory and usually preferred over txt or csv due to the sorting, formatting and calculation tools that it offers. It thus seemed appropriate to implement a streaming-enabled parser for these files.

For xlsx files (the most recent format) the data are stored as human-readable xml files in a zip archive, so that implementation of a streaming-enabled parser was relatively straightforward. The archive is first opened and metadata parsed to extract the sheet names (these information are kept in memory for later use). Blocs of xml data from the sheet to be read are then transferred to a web worker (equivalent to a separated thread) for parsing and the parsed rows sent back one by one to the main thread. On a regular desktop computer, parsing of an xlsx file of about 16 MB, corresponding to a single worksheet file of over 250,000 rows with an unzipped size of 180 MB, was completed in about 5 seconds, which we considered satisfying.

Older spreadsheet files (.xls) are unfriendly binary files of varying structures depending on their version. A deep dive into the file specifications was necessary (https://docs.microsoft.com/en-us/openspecs/office_file_formats/ms-xls), but ultimately it was possible to implement a parser that could read xls files dating from as far as 1995. A strategy similar to xlsx files was used here: a first parsing digests the structural information to extract the sheet names and byte offsets of the sheet data-streams in the file (these metadata are kept in memory to speed-up future access). The desired sheet data-stream is then transferred to a web worker, parsed and the resulting rows sent back to the main thread. With a regular desktop computer, parsing of a 17 MB file containing a single sheet of 65,536 rows (the maximum allowed for xls files) was completed in less than 3 seconds. Both spreadsheet parsers are able to extract results from formulas but will ignore dates (return them as numbers) and formatting applied to the numbers or texts.

### Column mapping

When attaching data files to the layout as *Results* or *Definitions*, *PlateEditor* allows the mapping of specified columns to a well name (*Well ID*) or a plate name/index (*Plate ID*) (**Fig 2A**). Mapping of a *Well ID* is required for result files but both *Well ID* and *Plate ID* are optional for definition files. Fetching of the data will differ depending on this mapping configuration. This is quite straightforward for results as the values will always be fetched at the desired well locations in the file. If a *Plate ID* field is provided, values for the well ID matching the desired plate name will be used. Otherwise, the number of times the desired well name occurs in the file will be used as a key to match the desired plate index. Things are more complex for *Definitions* due to the higher number of cases to consider. All four possible mapping configurations and the corresponding rules used to resolve the name of a given range index are summarized in **Table 1**. If the name cannot be resolved, the generic name of the item is used instead: *RangeName* (#*i*), where *RangeName* is the name given to the range and *i* the index of the item in the range. For display in the Analysis tool, in cases where the range item is tagged over multiple wells, the name resolved at the first well location (*i*.*e*. well with the lowest index) will be used.

**Table 1:** Rules used for the resolution of name for a given range index from a *Definition* file, based on the mapping configuration.

Columns mapped	Rules
**-**	Names are fetched row-by-row, as they appear in the file. For a range with *n* items, fetch the name at row *i* + (*n* × *j*), where *i* is the desired range index and *j* the desired plate index.
**Well ID**	Fetch the name that match the *j*^th^ occurrence of the well ID, where *j* is the desired plate index.
**Plate ID**	Fetch the name that match the *i*^th^ occurrence of the plate ID, where *i* is the desired range index.
**Well ID & Plate ID**	Fetch the name that match the desired well ID and plate ID

### Customization of range numbering

**Fig 5:**
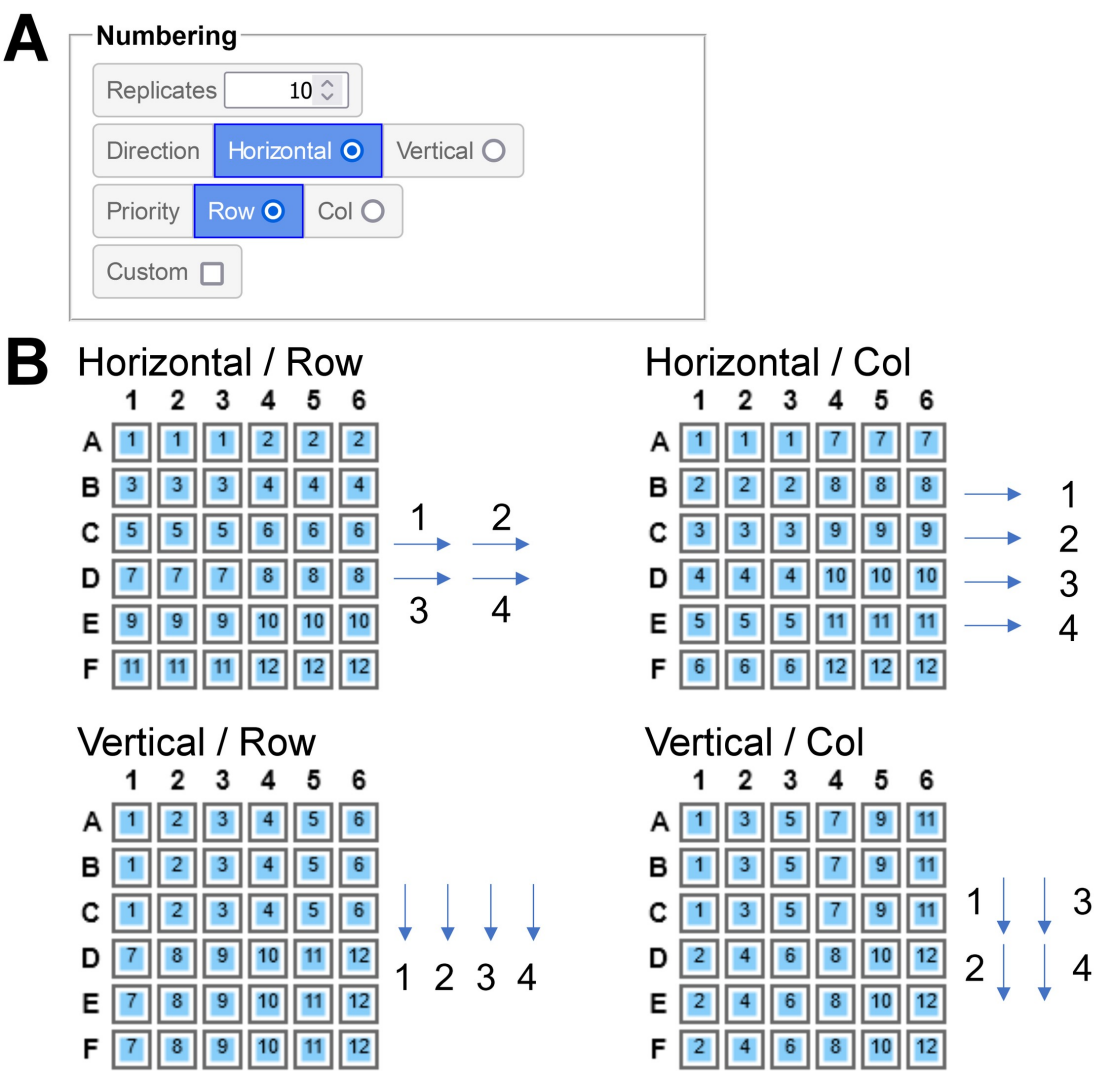
Customization of range numbering. **A**. Options available for the automatic numbering of Ranges. **B**. Possible numbering outcomes for each available Direction / Priority configuration, for a range of 3 replicates tagged in a bloc of 6×6 wells. A schematic indicating the numbering strategy for the four first range items is shown. Arrows indicate the 3 replicated wells of each range item.

In *PlateEditor Ranges* are self-incrementing *Areas* that can be tuned to match the desired numbering pattern. Several options are provided for automatic numbering (**Fig 5A**), giving the user the choice over the number of replicated wells per range index, the direction of the replication (Horizontal, *i*.*e*. left to right / Vertical, *i*.*e*. top to bottom) and whether Columns or Rows should be given the priority when numbering. The possible numbering outcomes for each option configuration is shown in **Fig 5B**. For specific cases that would not be covered by these options and to give the user more flexibility, a full Custom mode is also available. In this mode, the range index is manually indicated by the user through a prompt that displays after the wells have been tagged. In particular, this allows the preparation of layouts where range items occupy blocs of wells spanning multiple rows and/or columns.

## Plate layout examples

### Primary screening

**Fig 6:**
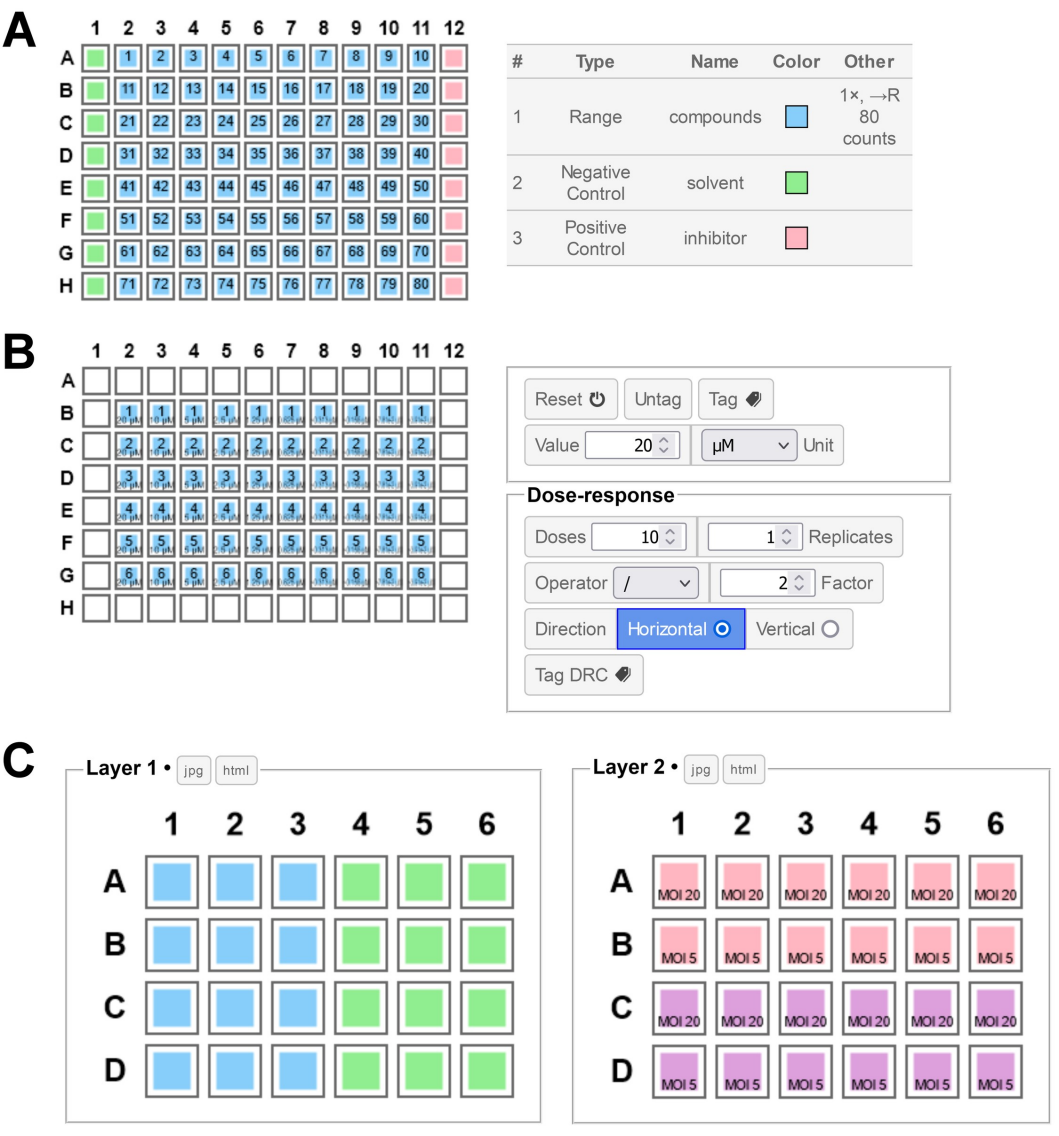
Plate layout examples. **A**. Example of layout for primary screening, defined on a single layer with a range (blue), a negative (green) and a positive (red) control. The list of Areas defined is shown on the right. **B**. Example of layout for a dose-response experiment. A single range with 10 replicates is sufficient here, along with concentrations data set using the configuration shown on the right. **C**. Combination experiment where 2 different type of cells (Layer 1, blue and green) are infected with 2 different virus strains (Layer 2, pink and purple) at 2 different multiplicity of infection (MOI, 5 and 20). *PlateEditor* automatically computes the 8 unique combinations when performing the data aggregation.

A layout for primary screening in 96-well plate can be created on a single layer, using 3 Areas: a range with one replicate that will represent the compounds, as well as a positive and a negative control (inhibitor and solvent, respectively. **Fig 6A**). The compound names can be resolved using a definition file attached to the range. A single result file holding well values for multiple plates can be attached as well. Result and definition plates can be paired by name using the auto-pairing option, which will allow them to remain synchronized. Values can be visualized for each plate in the form of heatmaps, making it simple to identify potential hit compounds. The *Controls* analysis tool will allow a rapid inspection of the Z-factor (Z’) for each plate. This example can be reproduced using the files available in the *Screening2* folder within the *Example* folder (the layout, definition and result files are included).

### Dose-response curves

In this example a batch of 6 compounds needs to be assessed by dose-response using a 96-well plate. The dose-response curve (DRC) should start with a 20 μM top concentration and contain 10 doses, with 2-fold dilutions between each doses. Preparation of the layout is straightforward: a single range with 10 replicates and its Direction and Priority properties set to their default value (Horizontal / Row) is first created, then tagged in the relevant wells. Using the same well selection a dose-response can be tagged automatically with the desired parameters, using the configuration as shown in **Fig 6B**. After binding of a result file, the *Grouped Analysis* tool can be configured with the concentration data (μM) as rows and the range as columns to display the desired dose-response table (as in **Fig 4**). A definition file can also be attached to the range in order to resolve the name of each compound. This example can be reproduced using the files available in the *DRC2* folder within the *Example* folder (the layout, definition and result files are included).

### Infection experiment (combinations)

In this example, two different cell types (CellA, CellB) should be engaged in viral infection experiments with two different viral strains (VirusA, VirusB) at two different multiplicity of infection (MOI, 5 and 20). Altogether 8 unique conditions should be tested (2 cells × 2 virus × 2 MOIs). Such layout can be prepared effortlessly by defining only 4 areas (2 for each cells and viruses) tagged on two independent layers (**Fig 6C**). MOI data can be added on the virus layer using the single concentration tagging tool to complete the layout. After binding of a result file, the *Column Analysis* tool will list automatically all the unique cell/virus/MOI combinations with their aggregated values and statistical summaries. This example can be reproduced using the files available in the *Infection* folder within the *Example* folder (the layout, definition and result files are included).

## Discussion

### Strength

From the start, *PlateEditor* was envisioned as a free in-browser solution allowing researchers to quickly design plate layouts and safely analyze their data from any computer. The strength of a web-based application is its broad availability and speed of deployment: users just need to connect to the web-page to enjoy the latest version and improvements without the need of regularly downloading and installing updates, which quickly becomes cumbersome when working with multiple computers. Such strategy is currently also adopted by technology companies (for example Google), which are delivering more and more common applications and tools through the web-browser. This allows users to work with multiple applications using a single software that is ubiquitous in all communication devices, from computers to smart-phones, including laptops and tablets. In our approach, we leveraged the power of JS to allow the application to run purely client-side, eliminating the constraints of uploading the data to the server for processing. This not-only reduces the time needed to access the file but also ensures complete privacy of the research data used within the application. Lastly, having no requirement for database or server connection and a minimum of dependencies, *PlateEditor* can easily be integrated into other workflows and software. We hope the availability of the source code will promote such usage and speed up the implementation of new features.

### Current limitations

Currently, *PlateEditor* offers only a limited support for tablets and smartphones, because the process of well selection requires drawing a selection box, which is not well supported by touchscreen device. Although all the functionalities are operational, the lack of support for this operation makes the preparation of layout tedious. The small screen size of smartphones would anyway make the use of 384-well plate layout (or bigger) quite uncomfortable, but a good usability of the application on tablets is still highly desirable. We are now considering solutions for this issue and hope to be able to implement them soon in the application. A related limitation is the lack of support for people with visual impairment. The need for well selection and the highly visual nature of the data representation and analysis using heatmaps and colors make it difficult however to implement work around.

Another limitation is the lack of support for writing and saving of big files by *PlateEditor*. The only feature currently impaired by this limitation is the *Push Layout* function, allowing layout data to be pushed to a result file. Since the file is generated in the memory, big files may pose an issue (browser stalling and crashing) so that this function is currently restricted to 5,000 rows. While reading is not a problem and web-browsers offer appropriate tools, access to the computer file system for writing is a clear vulnerability and is therefore restricted. Streaming client-side data in an output file is currently not possible in web-browsers without additional plugins or the use of experimental functions with limited support. To our knowledge, only one JS library currently implements such feature (StreamSaver.js, https://github.com/jimmywarting/StreamSaver.js) by installing a service worker online using GitHub static pages and using it to mimic a server sending the data back to the client in the form of a stream. We are currently evaluating the usefulness, safety and compatibility of this library and may add it to *PlateEditor* if deemed acceptable and necessary.

### Future updates planned

We are currently preparing additional analysis tools for *PlateEditor*. In particular, we are considering a tool for hits/outliers detection that could prove useful for the analysis of Primary screening data. We are also considering a tool to perform normalization using values from standard curves, in order to facilitate the treatment of data obtained from ELISA. This would further increase the usefulness and versatility of *PlateEditor* and warrant its implementation in more diverse workflows. In the near future, we also wish to upgrade *PlateEditor* with a graphical output tool that will allow the visualization of the aggregated data in graphs. Several types of graph are considered: bar graphs, dot plots and box plots (whiskers), along with basic tools to estimate the statistical significance between samples. A JS application for non-linear regression has already been deployed internally in our lab and its classes and functions will also be adapted to this graphical library when ready.
